# Vimentin Intermediate Filaments Maintain Membrane Potential of Mitochondria in Growing Neurites

**DOI:** 10.3390/biology13120995

**Published:** 2024-11-29

**Authors:** Alexander A. Dayal, Olga I. Parfenteva, Huiying Wang, Blen Amare Gebreselase, Fatima K. Gyoeva, Irina B. Alieva, Alexander A. Minin

**Affiliations:** 1 Institute of Protein Research, Russian Academy of Sciences, 119334 Moscow, Russiairina_alieva@belozersky.msu.ru (I.B.A.); 2 Belozersky Institute of Physical and Chemical Biology, Lomonosov Moscow State University, 119992 Moscow, Russia

**Keywords:** vimentin, intermediate filament (IF), mitochondria, mitochondrial membrane potential

## Abstract

Neural precursor cells contain two types of intermediate filaments (IFs): neurofilaments consisting of three IV type proteins and vimentin belonging to the type III IF proteins that disappear at the later stages of differentiation. The involvement of vimentin in neurogenesis was demonstrated earlier; however, the role of its temporary expression in neurons is not clear. We showed that the vimentin IFs that interacted with mitochondria maintained their membrane potential at the appropriate level, and thus, ensured their proper function. We examined the dependence of the mitochondrial membrane potential on the expression of vimentin in a CAD catecholaminergic neuronal cell line that was actively dividing in full culture media but stopped growing and started developing neurites when the serum was removed. Using the CRISPR Cas9 system to knock out the vimentin gene in these cells, we investigated the impact of this on the mitochondrial membrane potential. Our data show that the deletion of the vimentin IFs led to a decrease in the level of the mitochondrial potential. When the vimentin network in these cells was reconstituted by transfection with a plasmid that encoded human protein, the level of the potential was restored. Interestingly, mutated vimentin with a disrupted mitochondria-binding site had no such effect. Our data point to vimentin as a possible target in some neurological pathologies.

## Simple Summary

Vimentin, a type III protein of intermediate filaments (IFs) characteristic of mesenchymal cells, was found in neural cells, together with neurofilaments in the early stages of their differentiation or recovery after damage. Since in the fibroblasts, vimentin IFs were implicated in the maintenance of mitochondrial membrane potential, we decided to explore their possible impact on the mitochondria in neural cells. The CAD cell line offers a convenient model to investigate neuritogenesis, which is stimulated by the simple removal of serum from the culture medium. The results of this study show that, indeed, the deletion of vimentin in these cells led to a decrease in the membrane potential of mitochondria, though other IFs and neurofilaments remained. Furthermore, the restoration of vimentin IFs in the knockout cell line by the human vimentin caused an increase in the mitochondrial potential.

## 1. Introduction

Vimentin, a type III intermediate filament (IF) protein was found in almost all neural precursor cells. However, their differentiation into mature neurons is accompanied by the gradual replacement of vimentin IFs for neurofilaments, which are type IV IF proteins [1]. The studies from T. Shea’s laboratory demonstrated the involvement of vimentin IFs in the initial stages of neuritogenesis [2], though their exact role remains unclear. The observed localization of vimentin in the growing axons suggest its participation in the process of neuronal differentiation [3]. It was also shown that the depletion of vimentin in cultured neuroblastoma cells and hippocampal neurons suppressed the formation of neurites [2,4].

Mitochondria play an important role in neural differentiation, as well as in the functioning of mature neurons [5]. Being the main source of energy in the form of ATP, mitochondria are transported to and localized at the sites of energy consumption by the elaborate transport system based on microtubules and the actin cytoskeleton, together with associated motor proteins [6,7]. The formation and the maintenance of the dynamic structures of actin microfilaments and microtubules by themselves require a lot of ATP, not to mention the energy drain by the transport of different cargoes by motor protein ATPases [8]. This requires the reliable delivery to and functioning of mitochondria at appropriate sites. Remarkably, the mitochondria positioning at growth cones and extensions is correlated with an active axonal elongation [9], and the potential of mitochondria localized in the distal parts of neurites was found to be more hyperpolarized than that in the soma and along the main shaft [10].

By now, evidence has accumulated showing that IFs influence the shape; motility; and, more importantly, functioning of mitochondria in different cell types [11,12]. Neurons are no exception: the interaction of mitochondria with neurofilament proteins was extensively studied [13] and the involvement of this cytoskeleton component in mitochondrial functions was reliably proven. Furthermore, the available data suggest that the binding of mitochondria to neurofilaments in axons and dendrites is under the control of different regulatory signals [14,15]. At the same time, it is unknown whether the neurofilaments responsible for mitochondrial distribution regulate their functions, in particular, the mitochondrial membrane potential. Having shown that the vimentin IFs in fibroblasts were involved in the maintenance of the mitochondrial potential [16], we assumed that in developing neurons, this protein can perform a similar role. To test this hypothesis, we analyzed the mitochondrial membrane potential in the catecholaminergic neuronal cell line CAD, which offers a convenient model to study neurogenesis. These cells actively divide in full media but start neuronal differentiation when transferred to a serum-free medium [17]. The results of this work demonstrate that the knockout of the vimentin gene in CAD cells using the CRISPR Cas9 system led to a decrease in the mitochondrial membrane potential in nascent neurites. However, the reconstitution of vimentin IFs by the transfection of these cells with a plasmid encoding human vimentin caused the recovery of the mitochondrial potential to the initial level.

## 2. Materials and Methods

### 2.1. Cell Culture

CAD cells (CATH.a, CRL-3595, ATCC) were cultured in DMEM/F12 (PanEco, Moscow, Russia) supplemented with 10% fetal bovine serum (Biolot, Moscow, Russia), penicillin (100 µg/mL), and streptomycin (100 µg/mL) (Sigma, St. Louis, MO, USA) at 37 °C in a humidified atmosphere with 5% CO_2_. Differentiation was induced by switching the proliferating cells to a serum-free medium DMEM/F12. For microscopy, the cells were seeded on sterile coverslips and incubated for 24 or 48 h.

### 2.2. Gene Knockout

To knock out the vimentin gene in the CAD cells, the CRISPR Cas9 system was used to induce nonhomologous end joining after double-strand breaks. The plasmid vector pSpCas9(BB)-2A-Puro [18] (Addgene, Watertown, MA, USA) was digested by BstV2I (Sibenzyme, Novosibirsk, Russia), and the duplex was provided by two annealed oligos: CACCGCGCCAGCAGTATGAAAGCG and AAACCGCTTTCATACTGCTGGCGC, which were inserted into the sequence encoding the guide RNA to provide a pSpCas9(BB)-2A-Puro-Vim plasmid. The cells were transfected with this plasmid and a selection of the CAD(Vim-/-) cells was performed in the DMEM/F12 medium containing 2 µg/mL puromycin and 1 µg/mL verapamil.

### 2.3. Transfection

The transfection of cells with plasmids pVim(wt), Vim(P57R) [19], pSpCas9(BB)-2a-puro-Vim, and pVB6-Chromobody [20] was conducted using the Transfectin reagent (Evrogen, Moscow, Russia). Briefly, 1 µg of plasmid DNA was mixed with 1 µL of the transfection reagent in 0.1 mL of DMEM without serum and antibiotics and added to cells in 1 mL of complete DMEM/F12 medium.

### 2.4. Fluorescent Microscopy of Live Cells

Mitochondria in CAD cells were stained with the membrane potential sensitive dye JC-1 (0.1–2.0 µg/mL) (Molecular Probes, Eugene, OR, USA) [13,21] for 30 min at 37 °C. Following incubation, coverslips with cells were placed in a sealed chamber that contained DMEM/F12 medium and imaged using a Keyence BZ-9000 microscope (Itasca, IL, USA), which was equipped with an incubator for live-cell imaging. The temperature within the incubator was maintained at 36 ± 2 °C. The imaging was conducted with a PlanApo 63x objective and a 12-bit digital CCD camera. The acquired images were transferred to a computer using BZ II Viewer 1.41 software (Keyence, Itasca, IL, USA) and saved as 12-bit graphic files for further analysis.

JC-1 [5,5′,6,6′-tetrachloro-1,1′,3,3′-tetraethyl-benzimidazolylcarbocyanine iodide] is a lipophilic cationic dye that forms potential- and concentration-dependent J-aggregates. JC-1 in its monomeric form exhibits a green color (525 nm) when excited at 490 nm, whereas J-aggregates exhibit a red color (590 nm) when excited at 490 nm. Depending on the level of membrane potential and the concentration of the dye in the medium, the JC-1 uptake into the mitochondrial matrix either reaches a high concentration and forms J-aggregates or remains monomeric when the concentration is low. We selected conditions that helped to distinguish highly energized mitochondria with a red color from green mitochondria with a lower potential.

Transfected cells with restored vimentin IFs were detected by the expression of VB6-Chromobody, which allowed for the identification of vimentin filaments in the live cells.

### 2.5. Immunofluorescence

To stain the IFs, the cells were fixed with methanol at −20 °C for 10 min. Indirect immunofluorescence was then performed using chicken polyclonal antibodies against vimentin (Poly29191, BioVitrum, Moscow, Russia), mouse monoclonal antibodies V9 against vimentin (Sigma, USA), and mouse monoclonal antibodies RMd020 against neurofilaments (Sigma, USA). FITC- and TRITC-conjugated anti-mouse secondary antibodies (Jackson, West Grove, PA, USA) were used for detection. Microphotographs were acquired using a Keyence BZ-9000 microscope (Itasca, IL, USA) with a PlanApo 63x objective and a 12-bit digital CCD camera (Keyence, Itasca, IL, USA). 

### 2.6. Immunoblotting

SDS-PAGE was conducted according to Laemmli’s method [22], followed by immunoblotting, as previously described [23]. Vimentin was detected with the rabbit polyclonal antibody RVIM-AT [24], neurofilaments with the monoclonal antibody RMd020 (Sigma, USA), alfa-tubulin with the monoclonal antibody DM1A (Sigma, St. Louis, MO, USA), and secondary anti-mouse and anti-rabbit antibodies conjugated to horseradish peroxidase (Jackson, West Grove, PA, USA). As a substrate for peroxidase, hydrogen peroxide and diaminobenzidine were used, which gave a brown color. Uncropped blots showing all bands are presented in the Supplementary Materials.

### 2.7. Evaluation of Mitochondrial Membrane Potential

In the cells stained with JC-1, the mitochondrial membrane potential was evaluated by counting the mitochondria with high potential that showed red fluorescence of J-aggregates in the conditions that allowed for distinguishing them from those with low potential. The cells were incubated with 1.0 µg/mL of JC-1 for 30 min. Using ImageJ 1.38 software, the mitochondrial contours were defined within the region of interest, and then the “analyze particles” plugin was used. The average number of mitochondria with a high potential per 10 µm of the neurite length was defined.

### 2.8. Statistical Analysis

The numbers of neurites per cell were determined by analyzing the phase contrast images of cells incubated in serum-free medium during 24 h and are presented as the mean values with standard errors. Processes shorter than 20 µm were not considered. Data on the highly energized mitochondria are presented as their mean number per 10 µm region of neurites with standard errors. To check for normality, the Shapiro–Wilk test was used. An F-test was used to check for homogeneity of the variance. The significance of the differences was estimated statistically by the paired-sample Student’s *t*-test.

## 3. Results

### 3.1. Production of CAD Cell Line with Knockout of Vimentin Gene

**Figure 1:**
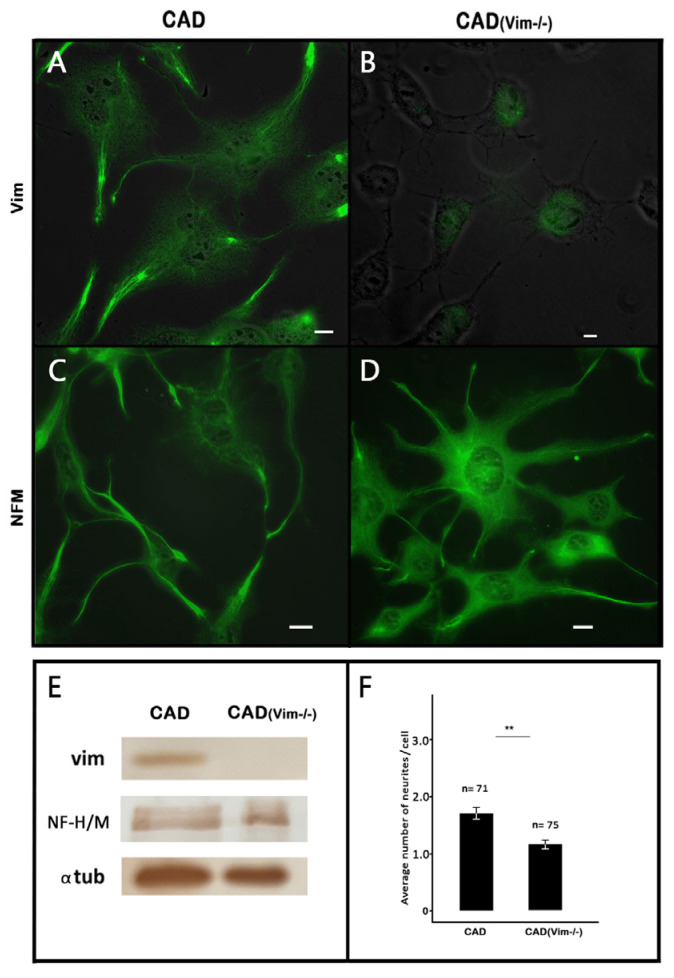
Deletion of vimentin IFs in CAD cells. Immunofluorescence of Vimentin IFs (**A**,**B**) and neurofilaments (**C**,**D**) in CAD (**A**,**C**) and CAD(Vim-/-) (**B**,**D**) cells. Fluorescence is overlaid with phase contrast images. Scale: 10 µm. (**E**) Western blot analysis of cell lysates using antibodies against vimentin, neurofilaments, and alfa-tubulin (as a loading control). (**F**) Average numbers of neurites per cell in CAD and CAD(Vim-/-) cell lines ± S.E., ** *p* < 0.05.

The catecholaminergic neuronal CAD cells represent a precursor cell line that undergoes neuronal differentiation when placed in a serum-free medium. Two types of IFs, vimentin and neurofilament triplet, are characteristic of these cells. It can be seen in Figure 1 that both networks were sparsely distributed in the cell bodies around the nuclei but showed increased density in the processes.

It was shown earlier [3] that vimentin IFs promote the outgrowth of neurites, though their exact role is still unclear. To better understand the contribution of the transitory expression of vimentin IFs in the formation of neuronal processes, we produced CAD cells with knockout of the vimentin gene and inspected their capacity to differentiate in a serum-free medium. Using the CRISPR Cas9 system, we obtained CAD(Vim-/-) cells completely devoid of vimentin, as shown in the results of the immunoblotting (Figure 1E and Figure S1) and fluorescence microscopy (Figure 1B). These cells, however, contained neurofilaments (Figure 1D) and preserved the ability to form neurites, though less effectively than the wild-type CAD cells (Figure 1F).

### 3.2. Vimentin-Null CAD Cells Contained Less Energized Mitochondria

We found earlier that vimentin IFs increase the membrane potential of mitochondria in fibroblasts [16]. Since the neurite extension of neural cells is an energy-consuming process, the energetic state of mitochondria is of great importance [5]. So, it could be supposed that a temporary expression of vimentin in developing neurons ensures the troubleproof function of mitochondria.

**Figure 2:**
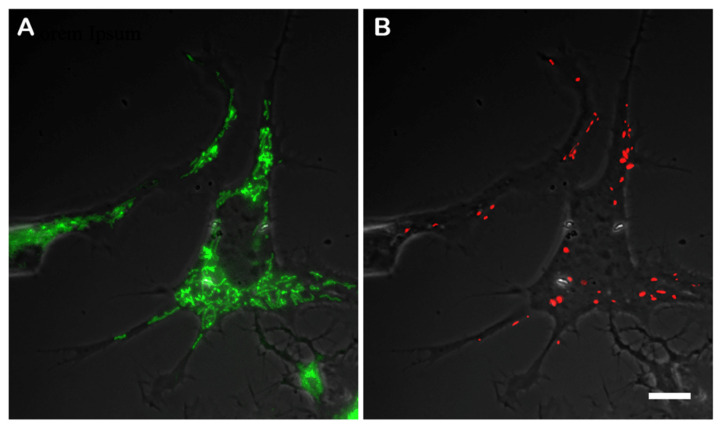
Mitochondria in CAD cells stained with 1.0 µg/mL of JC-1. Green fluorescence reveals mitochondria with lower potentials (**A**) and red shows those with higher potentials (**B**). Fluorescence is overlaid with phase contrast images. Scale: 10 µm.

To compare the membrane potential in the original CAD cells and the knockout CAD(Vim-/-) cells, we used the mitochondria-specific dye JC-1 to detect the highly energized mitochondria, as those that contained J-aggregates in the matrix had red fluorescence. The mitochondria with a lower membrane potential remained a green color because the concentration of the dye was low. However, the probability of achieving a sufficient concentration of JC-1 to form red J-aggregates depended on the concentration of the dye added to the medium. Almost all mitochondria had green staining when the cells were incubated with 0.1 µg/mL of JC-1, whereas only a few of them showed red staining. In contrast, most mitochondria acquired red fluorescence when the cells were treated with 2.0 µg/mL of JC-1. Thus, we chose the intermediate concentration of JC-1 as the most convenient for the quantification of the proportion of mitochondria with a higher potential. Figure 2 shows the CAD cells that were incubated with 1.0 µg/mL JC-1, which allowed for revealing the mitochondria with different levels of the potential. It is clearly seen that among the evenly distributed mitochondria with relatively low membrane potentials, those with a higher potential localized at the periphery of the cell and in the processes. Regarding the vimentin IFs in the differentiating CAD cells showed an increased density at the cellular periphery and in the processes (Figure 1A), it can be assumed that the mitochondria with higher potentials co-localized with the vimentin.

**Figure 3:**
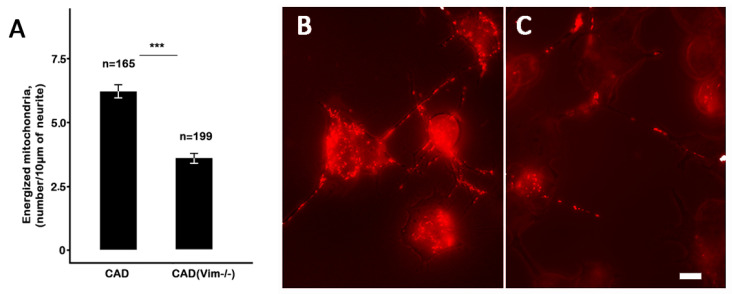
Neurites of the CAD cells contained more mitochondria with high potentials than those of the CAD(Vim-/-) cells. The data (**A**) present the mean numbers of highly energized mitochondria in the indicated number of neurite segments of 10 µm ± standard error; *** *p* < 0.001. Red fluorescence reveals the mitochondria with higher potentials in the CAD (**B**) and CAD(Vim-/-) cells (**C**). Scale: 10 µm.

The staining of the CAD(Vim-/-) cells with JC-1 demonstrated that the number of mitochondria with higher potentials was much lower than in the vimentin-containing cells, and such mitochondria were also localized in the processes more often than in the cell bodies. Figure 3 shows that in the cells that contained vimentin IFs, there were twice as much highly energized mitochondria than in the vimentin-null cells.

### 3.3. Recovery of Vimentin IFs in Knockout Cells Increased the Number of Energized Mitochondria

**Figure 4:**
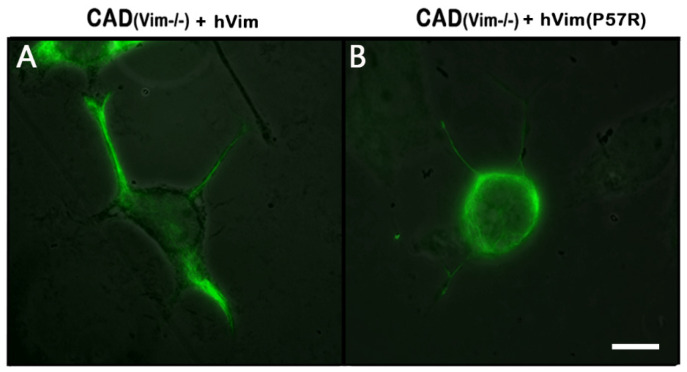
Reconstitution of vimentin IFs in CAD(Vim-/-) cells. Immunofluorescence of CAD(Vim-/-) cells expressing human vimentin (**A**) and CAD(Vim-/-) cells expressing human vimentin with mutation Vim(P57R) (**B**). Scale: 10 µm.

**Figure 5:**
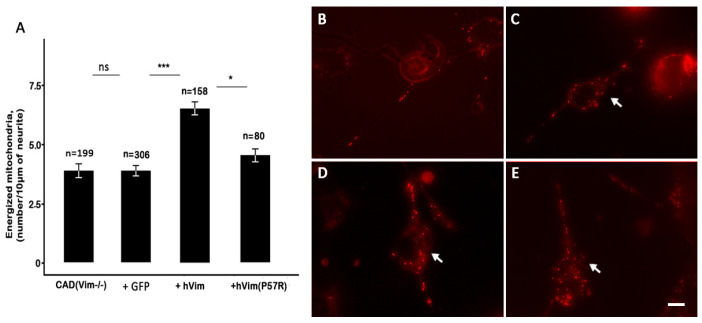
Neurites of the CAD(Vim-/-) cells that expressed human vimentin (+hVim) contained more mitochondria with high potentials than those of the control CAD(Vim-/-) or cells that expressed the VB6-Chromobody (+GFP) or mutated Vim(P57R) (+hVim(P57R). The data (**A**) present the mean numbers of highly energized mitochondria in the indicated number of neurite segments of 10 µm ± standard error; ns, not significant; * *p* < 0.05; *** *p* < 0.001. Red fluorescence reveals mitochondria with higher potentials in the CAD(Vim-/-) (**B**) and CAD(Vim-/-) cells transfected with the plasmid pVB6-Chromobody alone (**C**), pVim(wt) plus pVB6-Chromobody (**D**), or Vim(P57R) plus pVB6-Chromobody (**E**), and is indicated with arrows. Scale: 10 µm.

To make sure that the decrease in the mitochondrial potential in the CAD(Vim-/-) cells was, in fact, due to the absence of vimentin IFs, we transfected null cells with a plasmid encoding human vimentin, together with the Chromobody against vimentin, in order to detect the transfected cells. Figure 4A shows that the expression of recombinant protein fully restored the vimentin IFs in the CAD(Vim-/-) cells. The analysis of the mitochondrial membrane potential in the transfected cells showed that an expression of vimentin caused an increase in the number of mitochondria with high potential, in contrast to the cells that expressed the Chromobody alone (Figure 5). Interestingly, the expression of the mutated vimentin with the replacement of Pro-57 with Arg, which disrupted the vimentin’s capacity to bind to mitochondria [16], although it reconstituted the filament network (Figure 4B), did not affect the mitochondrial potential (Figure 5).

Thus, our data show that the vimentin played the role of the factor responsible for maintaining the mitochondrial potential at the high level in the growing neurites independently from the other IFs and neurofilaments.

## 4. Discussion

The results obtained in this study support our initial hypothesis that vimentin plays a regulatory role in the maintenance of mitochondrial membrane potential in neurons. We demonstrated that the vimentin IFs were necessary for maintaining a higher mitochondrial potential in the growing neurites, where the energy demands were high. The absence of vimentin led to a decrease in the mitochondrial potential in these neurites. Given that the vimentin and neurofilaments were co-expressed in the CAD cells, these results also suggest a unique role of vimentin in regulating the mitochondrial potential. These findings suggest that vimentin IFs are the integral factor for the regulation of the function of mitochondria within neurons, ensuring that energy production is efficiently matched to cellular needs.

Our findings provide further evidence that the vimentin IFs played a significant role in the mitochondrial dynamics, particularly in their ability to regulate the mitochondrial membrane potential and their distribution within neurons. Interestingly, previous research showed that the anchoring of mitochondria in neurons depends on the presence of a membrane potential and its interaction with neurofilaments, which are the major intermediate filaments (IFs) in neurons. Wagner and colleagues [13] demonstrated that isolated mitochondria anchored to neurofilaments in vitro only when they maintained a stable membrane potential. Under conditions when the mitochondrial membrane potential was dissipated by the addition of uncoupling agents, like valinomycin or FCCP, this anchoring was disrupted, and the neurofilaments detached from the mitochondria. This suggests that the interaction between IFs and mitochondria was closely tied to their bioenergetic status, particularly in the case of neurofilaments. Our study extended this concept to vimentin in CAD cells, where both neurofilaments and vimentin were co-expressed, indicating that vimentin may play a unique role in regulating mitochondrial function under conditions in which both types of IFs are present, such as during embryonic development or pathological states.

Furthermore, vimentin’s ability to regulate the mitochondrial potential in the presence of neurofilaments raises important questions about the functional division between these intermediate filaments (IFs) in neurons. While neurofilaments are critical for maintaining axonal stability, especially in mature neurons, our study suggests that vimentin plays a more dynamic role in mitochondrial regulation, particularly in regions where rapid energy production is required in the growing neurites. Previous research showed that fluctuations in the mitochondrial potential are closely associated with essential processes, such as synaptic activity and neuronal signaling [25]. Our findings indicate that vimentin may help to stabilize these fluctuations by ensuring that mitochondria with a high potential are preferentially localized to areas with high metabolic demands, thus supporting key neuronal functions more effectively.

Finally, our study also reinforced earlier findings showing that mitochondrial dysfunction, particularly disruptions leading to dissipation of their potential, is a critical factor in neurodegenerative diseases. Pathological conditions, such as amyotrophic lateral sclerosis (ALS) and Parkinson’s disease, are associated with impaired mitochondrial dynamics and neurofilament aggregation [26]. In light of our results, vimentin can offer a protective mechanism in neurons by supporting mitochondrial function.

The implications of the mitochondrial membrane potential in neuronal health and pathology are profound, as the threshold for its reduction that leads to apoptosis remains a critical point of investigation. Previous studies, such as those by Khodjakov and colleagues (2004) [27], showed that partial mitochondrial depolarization in rat substantia nigra neurons—which affected 10–15% of the mitochondria—did not immediately trigger apoptosis, even though mitochondrial damage occurred. Similarly, a reduction in the mitochondrial potential from 158 mV to 108 mV in rat cortical neurons did not appear critical for cell survival [28], while in other cell types, a partial reduction in the potential did lead to apoptotic signals [29]. These findings suggest that the heterogeneity of mitochondrial potential, both within single neurons and across cell populations, plays a role in determining the threshold for apoptosis.

In our study, we observed a significant drop in potential in the vimentin-depleted neurons, particularly in neurites where energy demands are high. This reduction might not necessarily lead to immediate cell death but could impair mitochondrial efficiency and overall neuronal function. As highlighted in previous research, fluctuations in the level of mitochondrial potential are a normal physiological phenomenon [30]; however, when these fluctuations are not properly regulated, such as in the absence of vimentin, the ability of neurons to sustain critical processes, like synaptic transmission and axonal transport, may be compromised. The observed potential drop in vimentin-depleted cells suggests that vimentin serves a protective role in maintaining mitochondrial function, particularly in regions of high metabolic demand. Without vimentin, mitochondria may be more susceptible to depolarization, which, if widespread or sustained, could lead to dysfunction or eventual cell death. This connection between the mitochondrial potential and pathology has broad implications for understanding neurodegenerative diseases, where mitochondrial dysfunction and IF dysregulation are common. The reduced mitochondrial potential in vimentin-null cells may parallel the mitochondrial impairments seen in conditions like Parkinson’s disease or amyotrophic lateral sclerosis (ALS), where mitochondrial bioenergetics are often compromised.

## 5. Conclusions

Altogether, this study provided evidence for the important role of vimentin IFs in growing neurons for maintaining the appropriate level of mitochondrial membrane potential to meet the energy supply requirements throughout the differentiation process.

## Data Availability

The data obtained during this study can be provided upon request.

## Supplementary Materials

The following supporting information can be downloaded from https://www.mdpi.com/article/10.3390/biology13120995/s1, Figure S1: Western blot analysis of lysates of CAD and CAD(Vim-/-) cells with antibodies against alfa-tubulin (A), neurofilaments (B), and vimentin (C). Images show entire filters with revealed protein bands.
