# Supplementary material for: Vimentin Intermediate Filaments Maintain Membrane Potential of Mitochondria in Growing Neurites
Source: Biology (Basel). 2024 Nov 29;13(12):995. doi: 10.3390/biology13120995 (PMC11726714; doi:10.3390/biology13120995)
Supplement: Supplementary file 1 [file biology-13-00995-s001.zip › biology-3332194-supplementary.pdf]

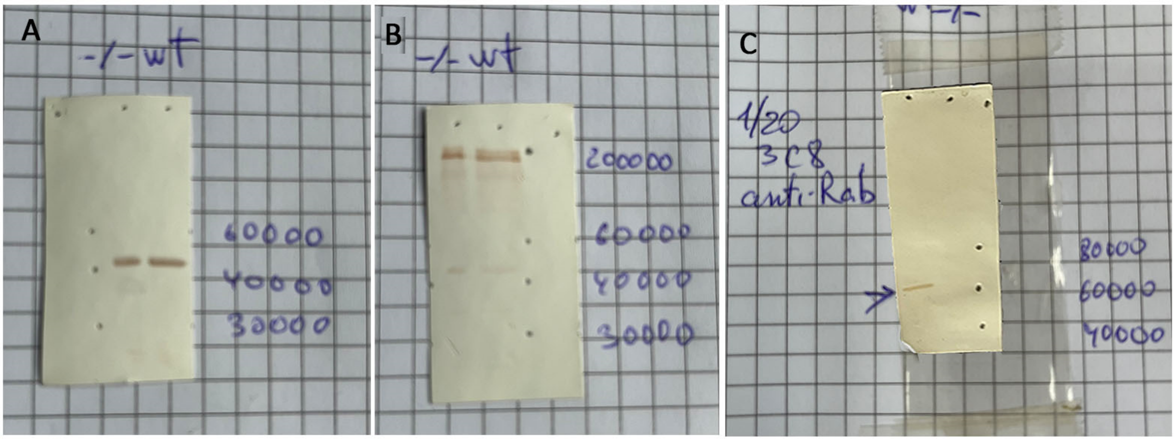

Figure S1: Western blot analysis of lysates of CAD and CAD(Vim-/-) cells with antibodies against alfa-tubulin (A), neurofilaments (B), and vimentin (C). Images show entire filters with revealed protein bands.
